# First case of mpox with monkeypox virus clade Ib outside Africa in a returning traveller, Sweden, August 2024: public health measures

**DOI:** 10.2807/1560-7917.ES.2024.29.48.2400740

**Published:** 2024-11-28

**Authors:** Carl-Johan Treutiger, Finn Filén, Moa Rehn, Johan Aarum, Andreas Jacks, Magnus Gisslén, Erik Sturegård, Maria Lind Karlberg, Oskar Karlsson Lindsjö, Klara Sondén

**Affiliations:** 1 Department of Infectious Diseases/Venhälsan, Södersjukhuset, Stockholm, Sweden; 2 Department of Communicable Disease Control and Health Protection, Public Health Agency of Sweden, Stockholm, Sweden; 3 Department of Microbiology, Public Health Agency of Sweden, Stockholm, Sweden; 4 Department of Communicable Disease Prevention and Control, Stockholm Region, Stockholm, Sweden; 5 Department of Infectious Diseases, Institute of Biomedicine, Sahlgrenska Academy at University of Gothenburg, Gothenburg, Sweden; 6 Department of Translational Medicine, Faculty of Medicine, Lund University, Malmö, Sweden; 7 Department of Medicine, Karolinska Institute, Stockholm, Sweden

**Keywords:** mpox, travel, public health, infection control, sequencing

## Abstract

An unprecedented upsurge in mpox, caused by clade I of the monkeypox virus (MPXV) was noted in Central Africa in 2024. The first mpox case with MPXV clade Ib outside Africa was reported in Sweden in mid-August. The case experienced a mild disease course after travelling to an affected country. No additional cases of this clade have been detected in Sweden. Strengthened public health measures and surveillance including whole genome sequencing are crucial to prevent establishment of novel MPXV clades.

On 14 August 2024, the World Health Organization (WHO) declared a public health emergency of international concern (PHEIC) in response to the current monkeypox virus (MPXV) clade I outbreak in Africa [1]. Here, we report the first mpox case of clade Ib outside of Africa in a Swedish traveller returning in August 2024 from an area in Central Africa affected by the current clade I outbreak.

## Case description

The case was a previously healthy man in his mid-30´s with no history of orthopoxvirus vaccination. During his travels from Sweden to Africa in August 2024, he first noticed a papular lesion in the genital area, occurring ca 13–14 days after a possible exposure. He suspected mpox, after being alerted by a person with whom he was in close physical contact in Africa and who had an mpox diagnosis.

The case’s general condition remained good, the papule umbilicated and ulcerated to a maximum diameter of 2 cm, and two smaller lesions appeared in the same area, with associated lymphadenopathy. Malaise and fever developed on day 4 after symptom onset, peaking at 38 °C. By day 6, a few new lesions (maximum diameter of 3 mm) appeared on his chest and forearms. The case had no medical consultation during the trip to Africa. 

Seven days after symptom onset, on the day of arrival in Sweden, the patient attended an outpatient clinic in Stockholm. By then, the fever had subsided, but the patient still experienced malaise. Real-time PCR for MPXV was performed on lesion swabs and returned a positive result on mid-August, and whole genome sequencing confirmed MPXV clade Ib the following day.

At a follow-up visit on day 11 after symptom onset, one new lesion (3 mm diameter) had appeared on one hand. The local lymphadenopathy had subsided. No respiratory or gastrointestinal symptoms were present, and no secondary or concomitant infections were diagnosed.

The case applied careful self-isolation from the appearance of the first lesion onwards, using a face mask and covering lesions when in public spaces. A travel companion developed mild throat symptoms shortly after arrival in Sweden and was monitored for 21 days, and tested negative for MPXV during this period. No secondary cases were identified, and the case recovered completely without complications. As the case only had contact with the travel companion after arrival in Sweden, no contact tracing was performed.

## Microbiological investigation and whole genome sequencing

The primary diagnosis was confirmed by real-time PCR at Karolinska University Hospital, Stockholm, Sweden. Furthermore, primary samples were sent to the Public Health Agency of Sweden (PHAS) for whole genome sequencing. Total nucleic acid was extracted using the MagLEAD 12gC robot with the kit MagDEA Dx SV (Precision System Science) following the manufacturer’s instruction after treatment of the samples by Proteinase K (Qiagen). The presence of DNA from MPXV was confirmed by RT-PCR using a method developed in-house at the PHAS.

Tiled amplification of the MPXV genome was performed using the NextGenPCR MPXV Amplification Primer Panel 96RXN (Molecular Biology Systems), containing two primer pools with primers designed by Martin Schou Pedersen (Department of Clinical Microbiology, Rigshospitalet, Copenhagen University Hospital, Copenhagen, Denmark and described by Welker et al. [2]). Five µl total nucleic acid of the extracted sample was mixed with Q5 HS master mix (New England Biolabs), 0.4 µM forward primer pool 1 or 2 in a final volume of 25 µL. The PCR programme was as follows: 95 °C 30 s, 25 cycles with 95 °C 15 s, 61 °C 2 min, 65 °C 5 min. The DNA concentration of the PCR products was quantified by Qubit 2.0 Fluorometer using Qubit dsDNA BR (Broad Range) Assay kit (ThermoFisher Scientific) [2].

Tiled PCR products were used to create a library using the Rapid Barcoding Kit (SQK-RBK114.96), which was then loaded onto an R10.4.1 PromethION flow cell according to the manufacturer’s instructions (Oxford Nanopore Technology (ONT)). The amplicon and the metagenomic libraries were loaded onto a R10.4.1 PromethION flow cells and sequenced on a PromethION instrument (ONT). MinKNOW software (version 23.07.12, ONT) was run in high accuracy mode.

Sequences were base called with Dorado (model dna_r10.4.1_e8.2_400bps_hac@v4.2.0) [3]. Minimap2 performed mapping towards the reference genome NC_003310.1, and following this, samtools was used for transformation into BAM and sorting, using a combined samtools_minimap2 docker [4-6]. For amplicon reads, the primer sequence was trimmed with samtools ampliconclip. The BAM file for metagenomic reads, the amplicon was combined, and a consensus sequence was generated using samtools concensus. Molecular typing was achieved using Nextclade, first towards the global hMPXV phylogeny and, based on placement there, towards the clade I phylogeny for increased resolution [7]. Additional data based on the GISAID repository were added to the phylogeny to identify the closest known relative [8].

## Phylogenetic analysis 

Phylogenetic analysis was performed to understand the genetic relationship of the isolate compared to previously sequenced isolates. Monkeypox virus clade I is divided into two subclades, Ia and Ib (Figure panel A). Subclade Ib branches into three main branches (Figure panel B). Notably, the Swedish case sequence (GISAID ID: EPI_ISL_19248512) is in the yellow branch of subclade Ib. Upon inspection of the branch containing the case sequence (Figure panel C), it is evident that the Swedish sequence is closely related to a sequence from Thailand (EPI_ISL_19350788), which was collected later in the same week. This proximity suggests a potential epidemiological link between the two cases. However, because of a lack of data, such a conclusion cannot be drawn at the time of publication.

**Figure:**
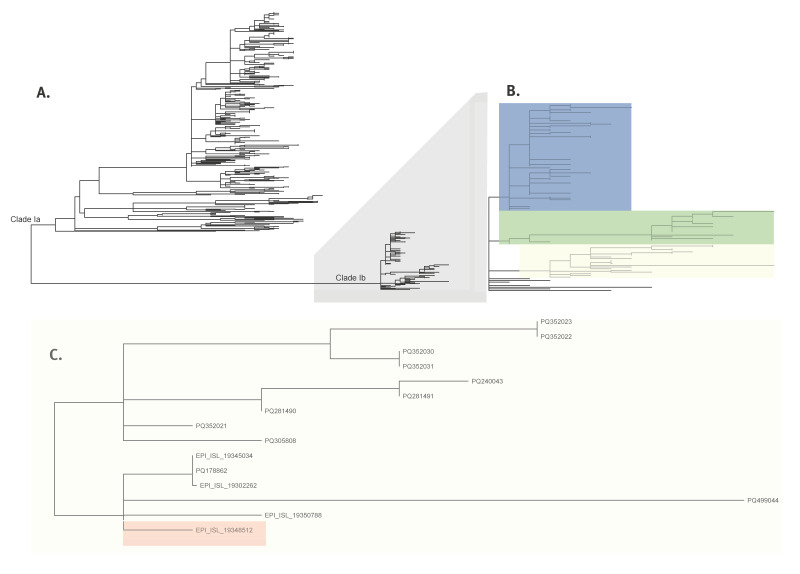
Phylogenetic analysis comparing monkeypox virus clade 1a and 1b sequences with the sequence from the mpox case, Sweden, August 2024

A. Clade I is divided into two distinct subclades, Ia and Ib (grey shading). The tree contains all good-quality sequences within Genbank.

B. Subclade Ib is divided into three branches with a number of spurious reads not belonging to any of the three major branches (blue, green and yellow shading). The sequence from the case in Sweden is situated in the yellow branch.

C. Magnification of the yellow branch shows the Swedish sequence (GISAID ID: EPI_ISL_19248512), indicated by pink shading, close to the sequence recovered from Thailand (GISAID ID: EPI_ISL_19350788), during the same week the Swedish sequence was sampled. Two samples from Kenya (EPI_ISL_19345034 and EPI_ISL_19302262) are from GISAID. Together with the sequences from Thailand and Sweden, these were all collected between the end of July and first half of August 2024. Sequences starting with PO are from the Nextclade guide-tree and created according to the Nextclade method, representing all good quality clade Ib sequences available within Genbank belonging to that branch of the tree.

## Public health measures

Since 2022, mpox is a notifiable disease in Sweden, subject to mandatory contact tracing and classified as a public health hazard. All detected cases must be reported to public health authorities. Suspected and confirmed cases are obliged to follow the rules of conduct deemed necessary by the treating physician to prevent further transmission of mpox from the notified case. At least eight regional laboratories dispersed throughout Sweden provide PCR analyses.

In response to the WHO’s declaration of PHEIC, as well as to prepare for potential further travel-associated cases of mpox caused by clade I and to mitigate possible impacts thereof, the PHAS urgently implemented several preventative measures including enhanced surveillance during the last 2 weeks of August 2024.

Travel recommendations, including advice for pre- and post-travel behaviour, were issued and public guidance material was prepared for display in arrival areas at airports. Vaccine protocols were updated to include recommendations for mpox vaccination before travel to areas with ongoing transmission of MPXV clade I, targeting individuals at high risk of exposure. The availability of vaccines and antivirals was reviewed. Furthermore, communication efforts targeting both the public and healthcare providers were initiated with the goal of increasing awareness of the disease, providing accurate information and reducing the risk of stigma. Guidelines for infection prevention and control in healthcare were reviewed and updated to ensure safe in- and outpatient care for mpox cases caused by any clade. 

Epidemic intelligence efforts were intensified, including more frequent monitoring of open sources, media and the scientific literature. Additionally, whole genome sequencing of MPXV was introduced at PHAS to detect potential clade I cases, which included all MPXV-positive samples at no cost to the regional health providers.

## Discussion

Mpox is an emerging zoonotic viral disease caused by MPXV, an orthopoxvirus. During 2024, an unprecedented number of suspected and ca 8,600 laboratory-confirmed [9] human cases of primarily MPXV clade I have been reported in the Democratic Republic of the Congo (DRC), with human-to-human transmission through close contact, including sexual contact [10]. The recently defined clade Ib has been detected in DRC and neighbouring countries, including community transmission reported from Burundi, Rwanda and Uganda. 

Although mild, this imported mpox case with clade Ib MPXV infection in Sweden highlights the cross-border threat of mpox and the role of international travel in its spread. Following identification of this case, additional mpox cases with MPXV clade Ib have been reported after travel in Africa from Thailand, India, Germany, the United Kingdom and the United States [9]. Since the case presented in Sweden, three new cases of mpox have been notified in Sweden and were determined to belong to MPXV clade II through whole genome sequencing. These three cases did not travel to an area with ongoing transmission of MPXV clade I nor reported any contact to a confirmed case of MPXV clade I. These cases reflect the continuous transmission of mpox in Europe in 2024.

## Conclusion

In response to the mpox case in Sweden and the WHO PHEIC, several public health measures were implemented in order to minimise the risk of further importation of cases. Mpox will likely continue to cause disease and the current situation highlights the importance of remaining vigilant to detect novel disease patterns and preventing further spread of clade Ib MPXV. 

## Supplementary Data

23000 Supplementary Table

## Supplementary Data

53000 Supplement disclaimer
