# Supplementary material for: First case of mpox with monkeypox virus clade Ib outside Africa in a returning traveller, Sweden, August 2024: public health measures
Source: Euro Surveill. 2024 Nov 28;29(48):2400740. doi: 10.2807/1560-7917.ES.2024.29.48.2400740 (PMC11605805; doi:10.2807/1560-7917.ES.2024.29.48.2400740)
Supplement: Supplementary Table [file 24-00740_SONDEN_Supplementary_table.pdf]

We gratefully acknowledge the following Authors from the Originating laboratories responsible for obtaining the specimens, as well as the Submitting laboratories where the genome data were generated and shared via GISAID, on which this research is based.

All Submitters of data may be contacted directly via www.gisaid.org

Authors are sorted alphabetically.

Accession ID	Originating Laboratory	Submitting Laboratory	Authors
EPI_ISL_19302262	National Public Health Laboratory - NVRL	Kenya Medical Research Institute (KEMRI) / Walter Reed Army Institute of Research - Africa	Gathii Kimita, Joseph K. Kaingu, George O. Awinda, Allan P. Lemtudo, Esther A. Omuseni, Josphat N. Nyataya, Beth K. Mutai, John N. Waitumbi
EPI_ISL_19345034	Arbovirology/Viral Haemorrhagic Fever Lab, Kenya Medical Research Institute (KEMRI)	Arbovirology/Viral Haemorrhagic Fever Lab, Kenya Medical Research Institute (KEMRI)	Langat,S., Nyunja,A., Pilarowski,G., Okunga,E., Ofula,V., Oluniyi,P., Koskei,E., Koka,H., Owaka,S., Chepkorir,E., Lutomiah,J., Langat,D., Khamadi,S. and Limbaso,K.
EPI_ISL_19348512	The Public Health Agency of Sweden	The Public Health Agency of Sweden	Oskar Karlsson Lindsjö, Maria Lind Karlberg, Klara Sondén
EPI_ISL_19350788	Hospital in Bangkok	Department of Medical Sciences, National Institute of Health	Pilailuk Okada; Siripaporn Phuygun; Nuttida Thongpramul, Titichaya Mebuathong, Pakorn Piromtong, Thanutsapa Thanadachakul, Thitipong Yingyong, Archawin Rojanawiwat, Ballang Uppapong, Yongyos Thammavuth
