# Supplementary material for: First case of mpox with monkeypox virus clade Ib outside Africa in a returning traveller, Sweden, August 2024: public health measures
Source: Euro Surveill. 2024 Nov 28;29(48):2400740. doi: 10.2807/1560-7917.ES.2024.29.48.2400740 (PMC11605805; doi:10.2807/1560-7917.ES.2024.29.48.2400740)
Supplement: Supplement disclaimer [file 24-00740_SONDEN_Supplement_disclaimer.pdf]

This supplementary material is hosted by Eurosurveillance as supporting information alongside the article "First case of mpox with monkeypox virus clade Ib outside Africa in a returning traveller, Sweden, August 2024: public health measures" on behalf of the authors who remain responsible for the accuracy and appropriateness of the content. The same standards for ethics, copyright, attributions and permissions as for the article apply. Eurosurveillance is not responsible for the maintenance of any links or email addresses provided therein.
